# Effects of Angiopoietin-1 on Hemorrhagic Transformation and Cerebral Edema after Tissue Plasminogen Activator Treatment for Ischemic Stroke in Rats

**DOI:** 10.1371/journal.pone.0098639

**Published:** 2014-06-04

**Authors:** Kunio Kawamura, Tetsuya Takahashi, Masato Kanazawa, Hironaka Igarashi, Tsutomu Nakada, Masatoyo Nishizawa, Takayoshi Shimohata

**Affiliations:** 1 Department of Neurology, Brain Research Institute, Niigata University, Niigata, Japan; 2 Department of Center for Integrated Human Brain Science, Brain Research Institute, Niigata University, Niigata, Japan; Massachusetts General Hospital/Harvard Medical School, United States of America

## Abstract

An angiogenesis factor, angiopoietin-1 (Ang1), is associated with the blood-brain barrier (BBB) disruption after focal cerebral ischemia. However, whether hemorrhagic transformation and cerebral edema after tissue plasminogen activator (tPA) treatment are related to the decrease in Ang1 expression in the BBB remains unknown. We hypothesized that administering Ang1 might attenuate hemorrhagic transformation and cerebral edema after tPA treatment by stabilizing blood vessels and inhibiting hyperpermeability. Sprague-Dawley rats subjected to thromboembolic focal cerebral ischemia were assigned to a permanent ischemia group (permanent middle cerebral artery occlusion; PMCAO) and groups treated with tPA at 1 h or 4 h after ischemia. Endogenous Ang1 expression was observed in pericytes, astrocytes, and neuronal cells. Western blot analyses revealed that Ang1 expression levels on the ischemic side of the cerebral cortex were decreased in the tPA-1h, tPA-4h, and PMCAO groups as compared to those in the control group (P = 0.014, 0.003, and 0.014, respectively). Ang1-positive vessel densities in the tPA-4h and PMCAO groups were less than that in the control group (p = 0.002 and <0.001, respectively) as well as that in the tPA-1h group (p = 0.047 and 0.005, respectively). These results suggest that Ang1-positive vessel density was maintained when tPA was administered within the therapeutic time window (1 h), while it was decreased when tPA treatment was given after the therapeutic time window (4 h). Administering Ang1 fused with cartilage oligomeric protein (COMP) to supplement this decrease has the potential to suppress hemorrhagic transformation as measured by hemoglobin content in a whole cerebral homogenate (p = 0.007) and cerebral edema due to BBB damage (p = 0.038), as compared to administering COMP protein alone. In conclusion, Ang1 might be a promising target molecule for developing vasoprotective therapies for controlling hemorrhagic transformation and cerebral edema after tPA treatment.

## Introduction

Thrombolytic treatment with tissue plasminogen activators (tPA) has been approved by the U.S. Food and Drug Administration as a standard treatment for cerebral infarction, and its therapeutic time window has recently been expanded to 4.5 h after onset [1]. While tPA treatment can be expected to greatly improve functional prognosis, administering it after its therapeutic time window can cause hemorrhagic transformation, exacerbate neurological symptoms, and even put the patient's life in danger [2]. Therefore, it would be beneficial to establish a vasoprotective treatment to prevent the hemorrhagic transformation of tPA treatment.

The hemorrhagic transformation that occurs after tPA treatment is thought to be caused by damage of the blood brain barrier (BBB). We previously demonstrated that tPA treatment after the therapeutic time window promoted expression of an endothelial cell–specific growth factor, vascular endothelial cell growth factor (VEGF), in BBB, MMP-9 activation, degradation of BBB components, and hemorrhagic transformation using a rat model of thromboembolic focal cerebral ischemia [3],[4]. Compared with tPA and control antibody, combination treatment with tPA and the anti-VEGF neutralizing antibody significantly attenuated VEGF expression in BBB, MMP-9 activation, degradation of BBB components, and hemorrhagic transformation, and it also improved motor outcome and mortality [3]. Therefore, inhibition of VEGF signaling pathway may be a promising therapeutic strategy for attenuating hemorrhagic transformation after tPA treatment.

Another endothelial cell–specific growth factor, angiopoietin-1 (Ang1) [5], is known to bind to the receptor Tie-2, which is expressed in various types of cells, such as endothelial cells, pericytes, and neuronal cells [6],[7]. Ang1 is known to participate in the survival of endothelial cells, vascular remodeling, and vascular maturation and stability [8],[9].

In addition, Ang1 has been reported to reduce postischemic vascular hyperpermeability that is triggered by VEGF [10]. However, it remains unknown whether hemorrhagic transformation after tPA treatment is related to the decrease in endogenous Ang1 after tPA treatment. Here, we hypothesized that administering Ang1 could attenuate hemorrhagic transformation and cerebral edema after tPA treatment by stabilizing blood vessels and inhibiting hyperpermeability. In this study, we performed tPA treatment on a rat thromboembolic model in order to confirm changes in Ang1 expression and to examine whether administering Ang1 would influence hemorrhagic transformation or cerebral edema.

## Materials and Methods

All operations concerning animal were performed according to ARRIVE (Animal Research: Reporting of In Vivo Experiments) guidelines [11].

### Animal model

This study was carried out in strict accordance with the recommendations in the Guide for the Care and Use of Laboratory Animals of the National Institutes of Health. The protocol was approved by the Niigata University Administrative Panel on Laboratory Animal Care (Permit Number: 36–4). All surgery was performed under inhalation anesthesia, and all efforts were made to minimize suffering.

The cerebral ischemia was created with the model of Okubo et al., in which the animals had their middle cerebral artery (MCA) blocked by autologous thrombi [3]. Specifically, male Sprague-Dawley rats (250–300 g body weight) (Tsukuba Breeding Center, Charles River Laboratories Japan, Inc., Ibaraki, Japan) were anesthetized through the inhalation of a mixture of 1.8% halothane, 30% oxygen, and 70% nitrous oxide. Rectal temperature was maintained at 37.0±0.5°C during surgery. With visualization aided with a surgical microscope, a midline incision was made in the anterior neck area to expose the left common carotid artery, the external carotid artery, and the internal carotid artery. Next, the external carotid artery was ligated, and a stump was severed. Then, an incision was made into the external carotid artery, and a catheter was inserted into the left internal carotid artery. A day ahead, 200 µL of the rat's own blood was mixed with 50 µL of thrombin and injected into a 380-µm-diameter polyethylene tube (PE20; BD, Franklin Lakes, NJ, USA). After storing it for 24–48 h at 4°C, thrombi that were 1 mm long were cut off and suspended in phosphate-buffered saline (PBS) containing 0.1% bovine serum albumin and then injected into the middle cerebral artery using a 580-µm-diameter polyethylene catheter (PE50; BD, Franklin Lakes, NJ, USA) for about 30 s in order to create a blockage (15 thrombi per animal).

### Cerebral blood flow measurement

Prior to vascular occlusion, the rats' skulls were exposed and 2-mm-diameter burr holes were created 2 mm posterior and 4 mm to the left of the bregma. Cerebral blood flow was measured before the surgery and 30 min after inducing the cerebral ischemia with a laser Doppler flowmeter (ALF21; Advance Co., Tokyo, Japan). The rats whose cerebral blood flow was less than 50% that before ischemia were excluded [3].

### Thrombolytic treatment with tPA

tPA was administered intravenously in the form of alteplase (a gift from Mitsubishi Tanabe Pharma Co., Osaka, Japan) at a dose of 10 mg/kg per animal. A PE50 catheter was inserted into the rats right inguinal veins; 10% of the dose was first administered as a bolus, and the remaining 90% was given continuously over 30 min. tPA treatment was performed 1 h after focal cerebral ischemia in the tPA-1h group and 4 h after in the tPA-4h group. The permanent middle cerebral artery occlusion (PMCAO) group did not receive tPA treatment, and the control group was not operated on at all. The mortality rates of the permanent ischemia group, tPA 1-hour group, and tPA 4-hour group were 17.4%, 6.7%, and 59.0% [3].

### Neurological evaluations

Neurological evaluations were conducted 24 h after the cerebral ischemia with a 6-point neurological scale [12]. Specifically, grade 5 indicated no neurological findings, grade 4 indicated an inability to move forward with the foot of the affected side, grade 3 indicated weak resistance to a force that was applied from the side on a level surface, grade 2 indicated turning to the affected side when pulled from behind on a level surface, grade 1 indicated spontaneously turning to the affected side, and grade 0 indicated an inability to move spontaneously or death.

### Measuring the volume of the cerebral infarct and edema

Twenty-four h after the cerebral ischemia, the subjects were given highly concentrated halothane and deeply anesthetized. After transcardiac perfusion with cold saline, the brains were extracted. The brains were cut into 3-mm slices and stained with a 2% 2,3,5-triphenyltetrazolium chloride solution (#264310; BD, Franklin Lakes, NJ, USA). After staining, the slices were photographed with a scanner (CanoScan LiDE 50; Canon Inc., Tokyo, Japan), and cerebral infarct volume and cerebral edema volume were measured with NIH Image J software 1.46r (National Institutes of Health, Bethesda, MD, USA) according to the method of Swanson et al [13]. These values were expressed as the proportion of the cerebral hemisphere occupied.

### Measuring the amount of cerebral hemorrhage

The amount of cerebral hemorrhage was measured as the amount of hemoglobin in all of the cerebral tissue with Drabkin's reagent (D5941; Sigma Aldrich, St. Louis, MO, USA) as reported previously [14]. Specifically, the entire cerebrum was homogenized with 3 mL of PBS and then centrifuged at 13,000 rpm for 30 min. Then, 0.4 mL of the supernatant was mixed with 1.6 mL of Drabkin's reagent and incubated at 56°C for 10 min. Finally, the absorbency was measured with an absorption spectrometer at 546 nm.

### Western blot

Twenty-four h after the cerebral ischemia, the subjects were euthanized with highly concentrated halothane, and transcardiac perfusion with cold saline was performed (3 animals from each group). The area of infarct was defined as the region showing neuronal cell loss with degenerated neuropil structure (spongiform appearance), and the non-infarct area surrounding the infarct area was defined as the peri-infarct area. The corresponding areas of the cerebral cortex were taken as samples. According to a previously reported method, the extracted brain tissue was homogenized by adding 7 times its weight of a cytolytic buffer solution containing 1% Triton X-100 (#9803; Cell Signaling Technology, Beverly, MA, USA) and inhibitors for proteases (P8340; Sigma-Aldrich, St Louis, MO, USA) and phosphatases (P2850 and P5726; Sigma-Aldrich, St Louis, MO, USA) [15], and centrifuging it at 14,000 rpm for 10 min. After measuring the protein concentration of the supernatant with the bicinchoninic acid method, 50 µg of protein was electrophoresed with Tris-glycine sodium dodecyl sulfate-polyacrylamide gel electrophoresis [16]. Next, this was transcribed onto a polyvinylidene fluoride membrane, and blocking was performed with 5% skim milk and 0.1% bovine serum albumin. The primary antibodies were the rabbit polyclonal anti-Ang1 antibody (AB10516: EMD Millipore Corporation, 1∶1,000) and rabbit polyclonal anti-phospho-Tie2 receptor antibody (AF2720: R&D Systems, Inc., 1∶1,000), which were reacted overnight at 4°C. After washing with PBS, horseradish peroxidase (HRP)-labeled anti-rabbit IgG antibody was reacted at room temperature for 1 h. Chemiluminescence was performed with chemiluminescent HRP substrate (EMD Millipore Corporation), and the desired protein band was then photographed with ImageQuant LAS4000 (GE Healthcare Japan, Tokyo, Japan). Actin was used as the internal control, and each protein band was quantified with a densitometer.

### Immunohistochemical staining

Twenty-four h after cerebral ischemia, the rats were euthanized with highly concentrated halothane, and transcardiac perfusion with cold saline, which was followed by perfusion with 4% paraformaldehyde, was performed. The extracted brains were fixed overnight in 4% paraformaldehyde that was dissolved in 20% sucrose. After methanol treatment, the samples were embedded in paraffin and then cut into 4-µm slices. After deparaffinization, the samples were reacted overnight at 4°C with a goat polyclonal anti-Ang1 antibody (sc-6319: Santa Cruz Biotechnology, Inc., 1∶200) and then stained using the Vectastain ABC kit (Vector Laboratories, Inc. Burlingame, CA, USA).

### Measuring Ang1-positive vessel density

The infarct area and peri-infarct area [4], which had been immunohistochemically stained with an anti-Ang1 antibody, and the cerebral cortex from a non-ischemic control brain were observed with an optical microscope at a magnification of 200×. Ang1-positive vessel density was calculated in all fields of view. The mean Ang1-positive vessel density was calculated from the values of 3 random fields of view from each sample. Each Group N = 9.

### Immunofluorescence staining and observation with a confocal microscope

The examination of Ang1 localization was performed according to a previously reported method of immunofluorescence staining that uses the free-floating method [16]. Twenty-four h after cerebral ischemia, the rats were euthanized, and transcardiac perfusion with cold saline, which was followed by perfusion with 4% paraformaldehyde, was performed. The brains were fixed with 4% paraformaldehyde. Next, the samples were cut into 50-µm slices with a vibratome (VT1000S; Leica Biosystems, Nussloch, Germany). Three-dimensional (3D) images were reconstructed with IMARIS imaging software (IMARIS 6.4.2; Bitplane AG, Zurich, Switzerland) from images that were taken at 0.23-µm intervals along the Z axis.

The primary antibodies that were used were a rabbit polyclonal anti-Ang1 antibody (ab93599: Abcam plc, 1∶100), mouse monoclonal anti-RECA1 antibody (MCA-970R: AbD Serotec, 1∶250), mouse monoclonal anti-glial fibrillary acidic protein (GFAP) antibody (3670: Cell Signaling Technology, Inc., 1∶250), goat polyclonal anti-platelet-derived growth factor receptor β (PDGFRβ) antibody (AF1042: R&D Systems, Inc., 1∶500), mouse monoclonal anti-microtubule associated protein (MAP2) antibody (M9942: Sigma-Aldrich Co. LLC, 1∶250), and rabbit polyclonal anti-FLAG antibody (F7425: Sigma-Aldrich Co. LLC, 1∶100). These were reacted overnight at 4°C and then stained with secondary antibodies, including Alexa Flour 488 and 568-conjugated IgG antibodies (Invitrogen, Carlsbad, CA, USA, 1∶1,000).

### Administration of COMP-Ang1 protein

Immediately before tPA administration in the tPA-4h group, 30 µg of the cartilage oligomeric protein (COMP)-Ang1 protein (ALX-201-314; Enzo Life Sciences, Inc., Farmingdale, NY, USA) that was dissolved in 200 µL of PBS was administered as a bolus through a catheter in the inguinal vein. Because recombinant Ang1 protein is poorly soluble, we used COMP-Ang1 protein, which has a higher solubility and is more active [17]. It is generated by replacing the N-terminal portion of the Ang1 protein with the coiled-coil domain of COMP [17]. As a control, 30 µg of COMP protein was similarly administered. The sample size was calculated before performing the experiments. We calculated the sample size needed to detect a difference in the amount of cerebral hemorrhage or cerebral edema volume between the COMP-Ang1 group and the COMP group with 80% power (α, 0.05; one-sided analysis; COMP-Ang1 group/COMP group  = 1.2). This calculation was based on the values obtained in our previous experiments [3]. A FLAG-tag was attached to the C terminus of the COMP-Ang1 protein, so that COMP-Ang1 protein localization could be investigated with immunohistochemical staining with an anti-FLAG antibody. The amount of cerebral hemorrhage, the volumes of the cerebral infarct and edema, and neurological evaluations were examined. These measurements were performed in a randomized and blind fashion.

### Statistical processing

All the data are presented as mean ± SEM. Differences in the parameters were analyzed using one-way analysis of variance (ANOVA) followed by Tukey's post hoc test or Mann-Whitney U test. Differences in the frequencies were assessed with Fisher's exact test. All the tests were considered statistically significant at P values <0.05.

## Results

### Localization of endogenous Ang1 in non-ischemic rat brains

**Figure 1:**
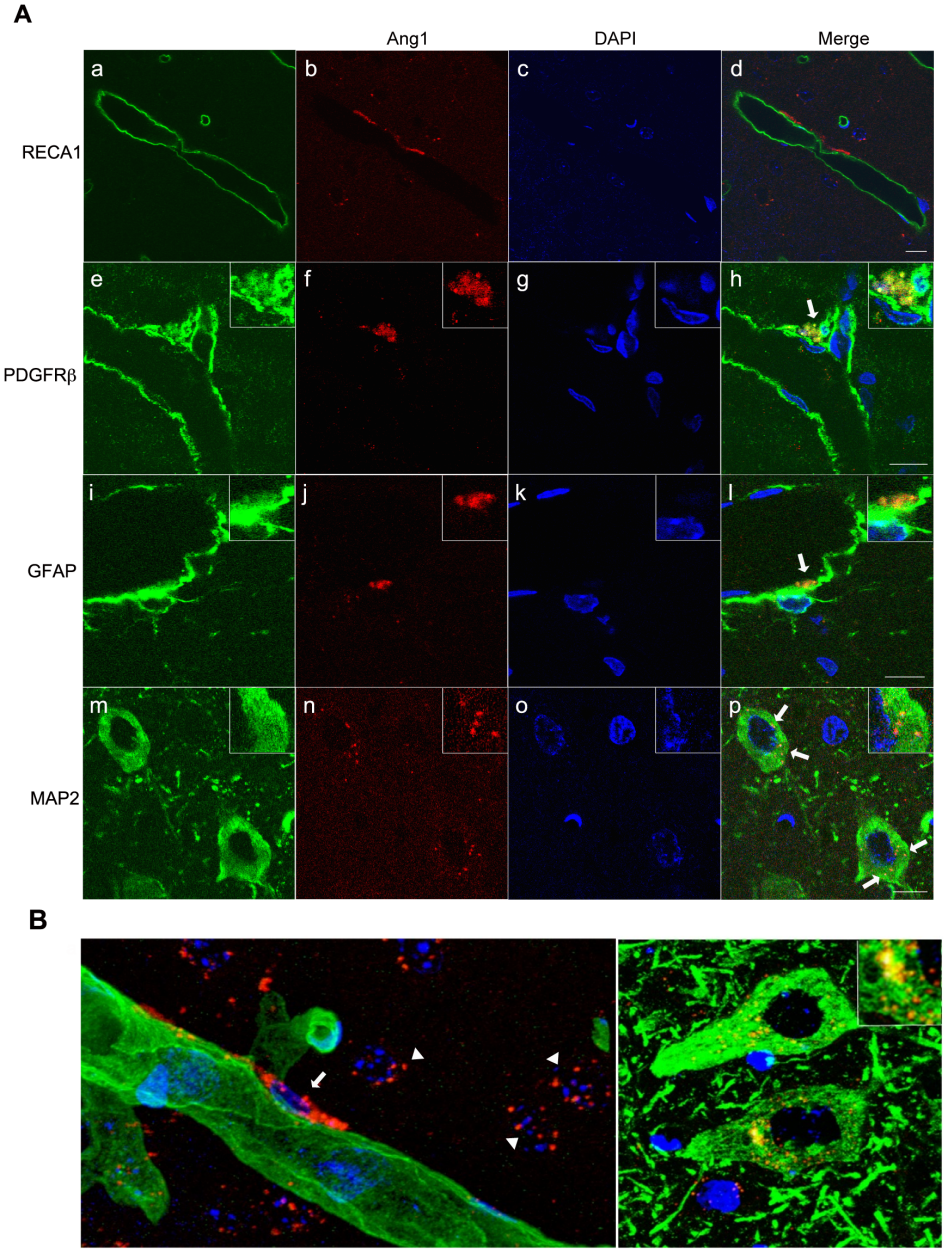
Endogenous Ang1 localization in non-ischemic rat brains visualized with confocal laser microscopy. (A) From left to right: markers of cells that make up the blood brain barrier (a, e, i, m; green), Ang1 (b, f, j, n; red), 4′,6-diamidino-2-phenylindole (DAPI) stain (c, g, k, o; blue), and a merged image (d, h, l, p). RECA1 is an endothelial cell marker protein; PDGFRβ is a pericyte marker; GFAP is an astrocyte marker; and MAP2 is a neuronal cell marker. RECA1, rat endothelial cell antigen; PDGFRβ, platelet-derived growth factor receptor β; GFAP, glial fibrillary acidic protein; MAP2, microtubule-associated protein 2; Ang1, angiopoietin-1. The scale bars are 10 µm. (B) Three-dimensional (3D) images of endogenous Ang1 in non-ischemic brains visualized with a confocal laser microscope. a: endothelial cell marker RECA1 (green) and endogenous Ang1 (red); b: neuronal cell marker MAP2 (green) and endogenous Ang1 (red). RECA1, rat endothelial cell antigen; Ang1, angiopoietin-1; MAP2, microtubule-associated protein 2. An arrow indicates Ang1 expressed in a pericyte. Arrowheads indicate Ang1 expressed in neuronal cells.

Immunohistochemical staining with the anti-Ang1 antibody was performed in order to clarify the localization of endogenous Ang1 in non-ischemic rat brains. Colocalization of endogenous Ang1 and the endothelial cell marker RECA1 was not observed, even though the endogenous Ang1 expression was observed on the outside of RECA1 (Figures 1Aa–d). However, the colocalization of endogenous Ang1 with the pericyte marker PDGFRβ (Figure 1Ae–h) and the astrocyte marker GFAP (Figure 1Ai–l) was observed. Moreover, the punctate colocalization of Ang1 with the neuronal cell marker MAP2 was observed on the cytoplasm of neuronal cells (Figures 1Am–p). In 3D images of RECA1 and endogenous Ang1, endogenous Ang1 expression was not observed in endothelial cells, but the expression of Ang1 is consistent with the localization pattern of pericytes (Figure 1Ba). In 3D images of MAP2 and endogenous Ang1, punctate endogenous Ang1 expression was observed in the cytoplasm of neuronal cells (Figure 1Bb). We did not note any signal when using only the secondary antibody (Figure S1).

### Effects of focal cerebral ischemia on endogenous Ang1 expression

**Figure 2:**
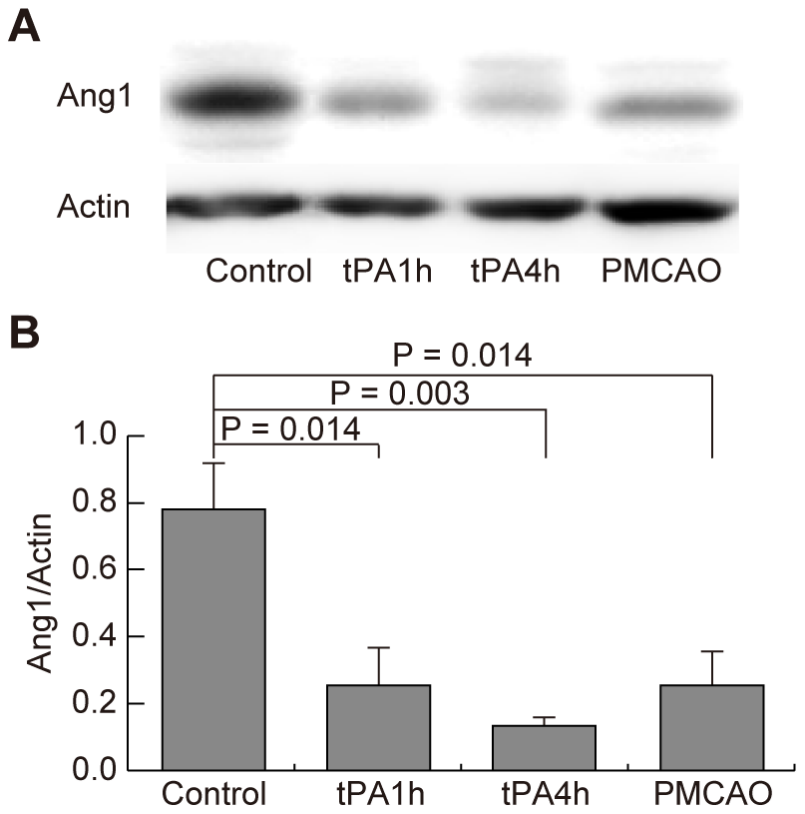
Changes in Ang1 expression due to focal cerebral ischemia. (A) Western blot analysis with an anti-Ang1 antibody. Each group N = 5. tPA, tissue plasminogen activator; PMCAO, permanent middle cerebral artery occlusion; Ang1, angiopoietin-1. (B) Measurements with a densitometer.

Changes in endogenous Ang1 expression 24 h after focal cerebral ischemia were investigated with Western blotting. Endogenous Ang1 expression levels on the ischemic side of the cerebral cortex were decreased in the tPA-1h, tPA-4h, and PMCAO groups as compared to that in the control group (P = 0.014, 0.003, and 0.014, respectively; Figures 2A, B). The Ang1 expression level in the tPA-4h group was the lowest among the ischemic groups, although there were no significant differences among these groups (Figure 2B). Protein fragments that would suggest limited degradation were not found.

### Effects of focal cerebral ischemia on endogenous Ang1-positive vessels

**Figure 3:**
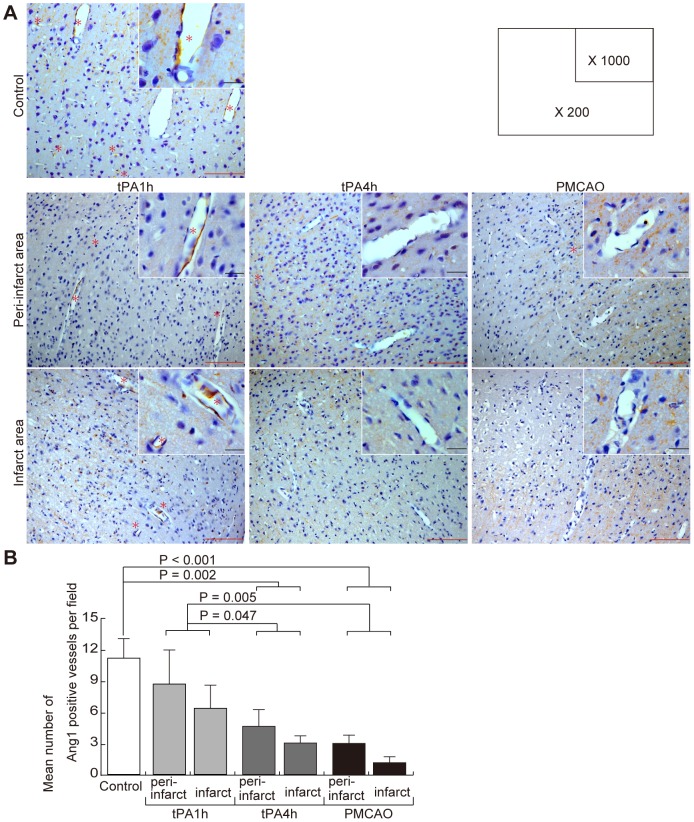
Changes in Ang1-positive vessels due to focal cerebral ischemia. (A) Immunohistochemical staining with an anti-Ang1 antibody. Representative findings are shown of the peri-infarct and infarct areas of the control, tPA-1h, tPA-4h, and PMCAO groups. High magnification (1,000×) is shown in the upper right of the low magnification (200×) photograph. Ang1-positive vessels were shown by asterisk. tPA, tissue plasminogen activator; PMCAO, permanent middle cerebral artery occlusion; Ang1, angiopoietin-1. The black scale bar is 10 µm, and the red scale bar is 100 µm. (B) Mean number of Ang1-positive vessels. Three locations were chosen randomly in the control cerebral cortex and in each infarct area and peri-infarct area. The figures are the mean number of Ang1-positive vessels from 3 random fields of view of an optical microscope at 200× magnification. Each group N = 5.

Because endogenous Ang1 expression was observed in neuronal cells in addition to cerebral blood vessels (Figure 1Am–p), immunohistochemical staining was used to quantify endogenous Ang1-positive vessel density 24 h after focal cerebral ischemia with the goal of evaluating endogenous Ang1 expression in cerebral blood vessels (Figure 3A). Endogenous Ang1-positive vessel density in the tPA-1h group was not reduced as compared to that in the control group (p = 0.105), while those of the tPA-4h and PMCAO groups were reduced in comparison to that of the control group (p = 0.002 and <0.001, respectively) (Figure 3B). In addition, endogenous Ang1-positive vessel densities in the tPA-4h and PMCAO groups were lower than that in the tPA-1h group (p = 0.047 and 0.005, respectively) (Figure 3B).

### Incorporation of COMP-Ang1 protein into pericytes

Next, in order to investigate whether administering Ang1 protein would be effective against BBB damage after tPA treatment, Ang1 protein was intravenously administered immediately before tPA administration in the tPA-4h group. In order to confirm the effects of COMP-Ang1 protein on the Ang1-Tie2 receptor signaling pathway, COMP-Ang1 protein was administered intravenously at doses of 5 µg, 10 µg, and 30 µg. Twenty-four h later, Western blotting was performed on these cerebral cortical samples to quantify the levels of expression of phospho-Tie2 receptors, which reflect Ang1 receptor activation. Because the only 30-µg dose resulted in increased phospho-Tie2 receptor expression compared to brains that did not receive COMP-Ang1 (data not shown), this dose was used for the following experiments.

**Figure 4:**
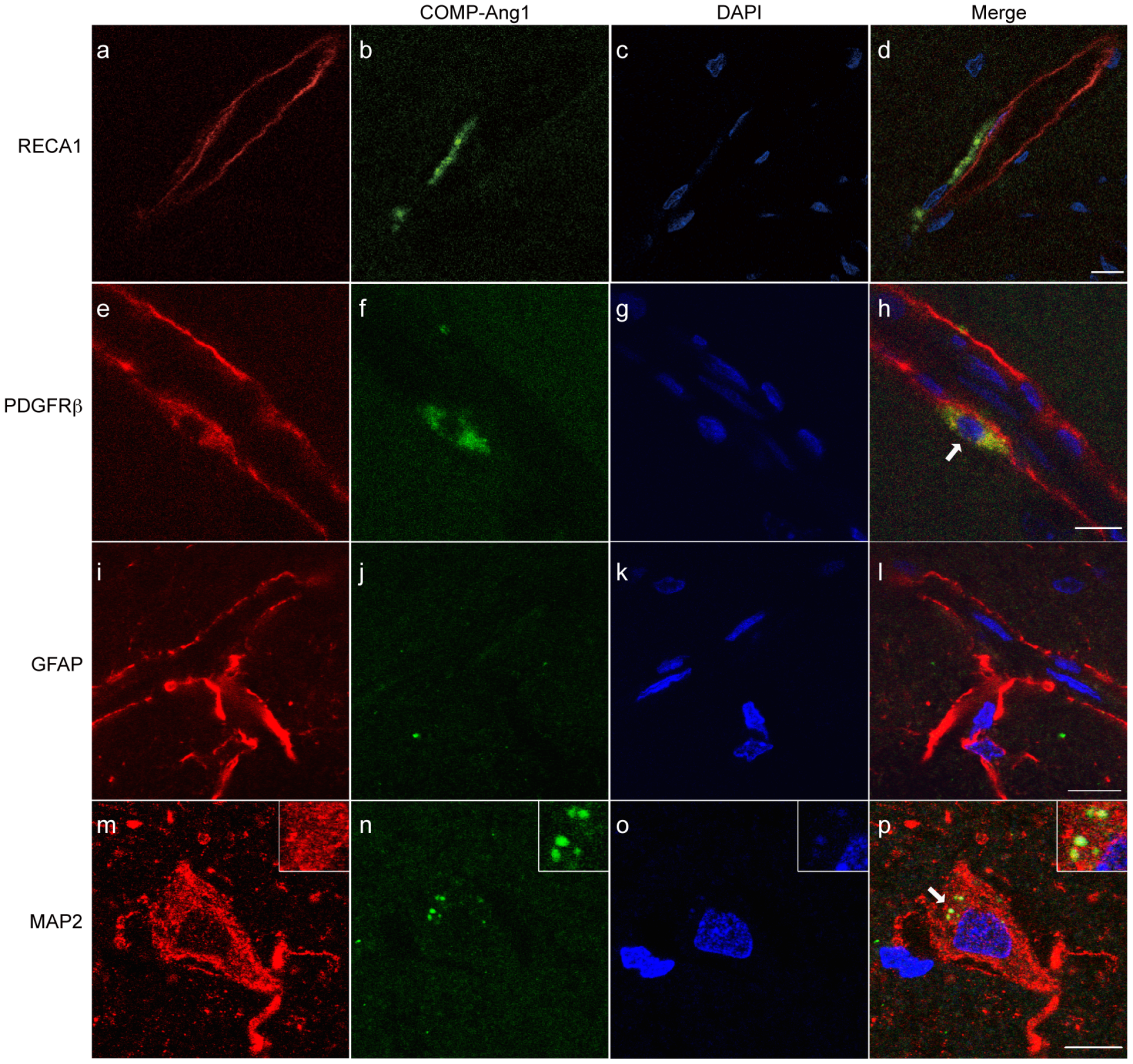
COMP-Ang1 protein localization in the tPA-4h group visualized with a confocal laser microscope. From left to right: markers of cells that make up the blood brain barrier (a, e, i; red), neuronal cell (m; red), COMP-Ang1 protein (b, f, j, n; green), DAPI stain (c, g, k, o; blue), and a merged image (d, h, l, p). Immunostaining with an anti-FLAG antibody was performed on COMP-Ang1 proteins in order to differentiate them from endogenous Ang1. RECA1 is an endothelial cell marker protein; PDGFRβ is a pericyte marker; GFAP is an astrocyte marker; and MAP2 is a neuronal cell marker. RECA1, rat endothelial cell antigen; PDGFRβ, platelet-derived growth factor receptor; GFAP, glial fibrillary acidic protein; Ang1, angiopoietin-1. The scale bars are 10 µm.

In addition, immunofluorescent staining was performed in order to confirm whether the COMP-Ang1 protein was reaching the microvessels and neuronal cells of the infarct area and the peri-infarct area at the 30-µg dose. In the tPA-4h group, anti-FLAG antibody-stained COMP-Ang1 protein mainly colocalized with the pericyte marker PDGFRβ in the peri-infarct area (Figure 4Be–f), but colocalization was not observed with the endothelial cellular marker RECA1 (Figure 4Ba–d) and the astrocyte marker GFAP (Figure 4Bi–l). Incorporation of COMP-Ang1 protein into neuronal cells was observed in peri-infarct area (Figure 4Bm-p), but not in infarct area (data not shown). We did not note any signal when using only the secondary antibody (Figure S2).

### Effects of COMP-Ang1 protein administration on hemorrhagic transformation and cerebral edema after tPA treatment

**Figure 5:**
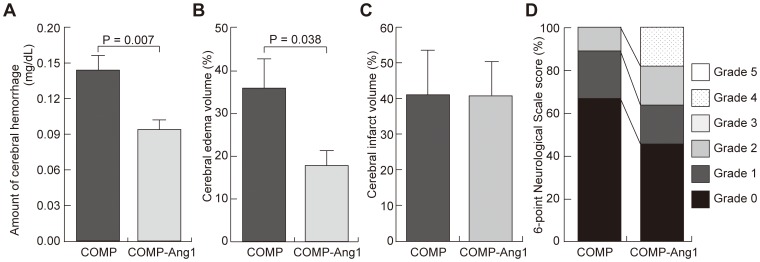
Effects of COMP-Ang1 protein administration on the tPA-4h group. These panels show the amount of cerebral hemorrhage (A), cerebral edema volume (B), cerebral infarct volume (C), and the prognosis with a 6-point neurological scale score (D) 24 h after ischemia. A-C were performed on the COMP-Ang1 group (N = 5) and the COMP group (N = 5). D was performed on the COMP-Ang1 group (N = 11) and the COMP group (N = 9). Cerebral edema and infarct volumes are expressed as proportions on the ischemic side of the cerebral hemisphere. The amount of cerebral hemorrhage is expressed as hemoglobin concentration in a whole cerebral homogenate. COMP; cartilage oligomeric protein, COMP-Ang1; cartilage oligomeric protein-angiopoietin-1.

In order to confirm the effects of administering COMP-Ang1 protein on the hemorrhagic transformation and the cerebral edema that occur after tPA treatment, a comparison was made between the tPA-4h group, which received 30 µg of COMP-Ang1 protein, and a control group given 30 µg of COMP protein and tPA at 4 h after ischemia. In a whole cerebral homogenate, hemoglobin was shown to be significantly lower in the group that received the COMP-Ang1 protein (0.093 mg/dL vs. 0.144 mg/dL; p = 0.007) (Figure 5A). Cerebral edema was significantly suppressed in the COMP-Ang1 group compared to the COMP group (17.8% vs. 35.9%; p = 0.038) (Figure 5B). No significant difference was observed between the groups for cerebral infarct volume (40.7% vs. 41.0%; p = 0.983) (Figure 5C). On the 6-point neurological score, grade 4 was only observed in the COMP-Ang1 group, and this group tended to have fewer grade-0 scores, but, overall, no significant differences were observed (p = 0.295) (Figure 5D).

## Discussion

This study first examined the expression of endogenous Ang1 in non-ischemic rat brain tissue, confirming its expression in pericytes, astrocytes, and neuronal cells. Previous studies have confirmed that endogenous Ang1 is produced in pericytes [18] and is expressed in astrocytes [19] and tumor cells [20]. These were consistent with the results of this study. Furthermore, while very small levels of expression have been reported in neuronal cells at the mRNA level [21], this study is the first to confirm the expression in neuronal cells at the protein level with cytoplasmic punctate localization.

Next, we demonstrated that endogenous Ang1 expression decreased after focal cerebral ischemia. A few studies have reported changes in Ang1 expression at the mRNA and protein levels after cerebral ischemia [19],[22],[23], but views on the matter are not settled. Furthermore, we could not find any studies that investigated changes in endogenous Ang1 expression after tPA treatment. By Western blotting, the ischemic group, especially the tPA-4h group, exhibited decreases in endogenous Ang1 expression 24 h after cerebral ischemia as compared to the control group. Because the hemorrhagic transformation that was observed in the tPA-4h group were due to BBB damage, we focused on the changes in endogenous Ang1 levels in the BBB by using immunohistochemical staining to examine the endogenous Ang1 expression in the BBB. Ang1-positive vessel density in the tPA-1h group, which had few hemorrhagic transformation, was not decreased compared to that in the control group, but a significant decline was observed in the tPA-4h group, which had many such transformation, compared to those in the control and tPA-1h groups. This finding suggests that decreased endogenous Ang1 expression plays a role in hemorrhagic events that occur when tPA is administered after the therapeutic time window. Because Ang1 plays a role in vascular stability [9], the decreased Ang1 level may have resulted in BBB damage. However, endogenous Ang1 expression did decrease in the PMCAO group, which did not have many hemorrhagic transformation [3]. This suggests that hemorrhagic transformation is not only regulated by Ang1, but may also be dependent on a variety of factors, including tPA usage, VEGF [3], PDGF [24], and angiopoietin-2 as reported by other investigators [23].

In order to confirm that reduced endogenous Ang1 expression was involved in the hemorrhagic transformation after tPA treatment, COMP-Ang1 protein was administered concurrently with tPA treatment. We demonstrated that COMP-Ang1 protein could suppress the hemorrhagic transformation as well as cerebral edema after tPA treatment. Several studies have reported that the ability of Ang1 to suppress vascular hyperpermeability occurs by increasing glycocalyx in endothelial cells [25], acting on tight junction proteins [26], shrinking intercellular spaces [27], and by working through signaling by PDGF-B in pericytes [28]. Furthermore, this study demonstrated that the administered COMP-Ang1 protein colocalized with pericytes and not endothelial cells. Future studies need to confirm whether COMP-Ang1 could suppress hemorrhagic transformation and cerebral edema after tPA treatment by suppressing the permeability that is mediated by PDGF-B signaling in pericytes.

In contrast, this study demonstrated that COMP-Ang1 protein could not decrease infarct size, although a previous study reported its neuroprotective effect [10],[29]. Several reasons can be given for this lack of neuroprotective effects of COMP-Ang1 protein, which may include the timing and method of administration. Further studies need to be conducted whether the timing and method of administration of COMP-Ang1 protein may influence the neuroprotective effects.

The present study has a limitation that we could not quantify brain edema by wet-dry measurement or Evans blue leakage using *in vivo* studies. Further studies are required to determine the effects of COMP-Ang1 on cerebral edema after tPA treatment.

In conclusion, this study clarified the localization of endogenous Ang1 in pericytes, astrocytes, and neuronal cells in non-ischemic rat brain tissue. In addition, Ang1-positive vessel density decreased when tPA treatment for focal cerebral ischemia was performed after the therapeutic time window. Because administering COMP-Ang1 protein may result in the suppression of hemorrhagic transformation and cerebral edema, Ang1 can be considered to be a promising target molecule for vasoprotective treatment to prevent the BBB damage that accompanies tPA treatment.

## Supporting Information

Figure S1 **Negative controls for staining presented in** **Figure 1** **.** (A) Non-ischemic rat brain samples showing the absence of non-specific staining on using only goat secondary antibodies. From left to right: Alexa Fluor 568 goat anti-mouse IgG, Alexa Fluor 488 goat anti-rabbit IgG, 4′,6-diamidino-2-phenylindole (DAPI) stain, and a merged image. (B) Non-ischemic rat brain samples showing the absence of non-specific staining on using only donkey secondary antibodies. From left to right: Alexa Fluor 568 donkey anti-goat IgG, Alexa Fluor 488 donkey anti-rabbit IgG, DAPI stain, and a merged image.
(TIF)

Figure S2 **Negative controls for staining presented in** **Figure 4** **.** (A) Rat brain samples from cartilage oligomeric protein-angiopoietin-1 (COMP-Ang1) group showing the absence of non-specific staining using only goat secondary antibodies. From left to right: Alexa Fluor 568 goat anti-mouse IgG, Alexa Fluor 488 goat anti-rabbit IgG, 4′,6-diamidino-2-phenylindole (DAPI) stain, and a merged image. (B) Rat brain samples from COMP-Ang1 group showing the absence of non-specific staining using only donkey secondary antibodies. From left to right: Alexa Fluor 568 donkey anti-goat IgG, Alexa Fluor 488 donkey anti-rabbit IgG, DAPI stain, and a merged image.
(TIF)
