# Supplementary figures and images for: Effects of Angiopoietin-1 on Hemorrhagic Transformation and Cerebral Edema after Tissue Plasminogen Activator Treatment for Ischemic Stroke in Rats
Source: PLoS One. 2014 Jun 4;9(6):e98639. doi: 10.1371/journal.pone.0098639 (PMC4045756; doi:10.1371/journal.pone.0098639)

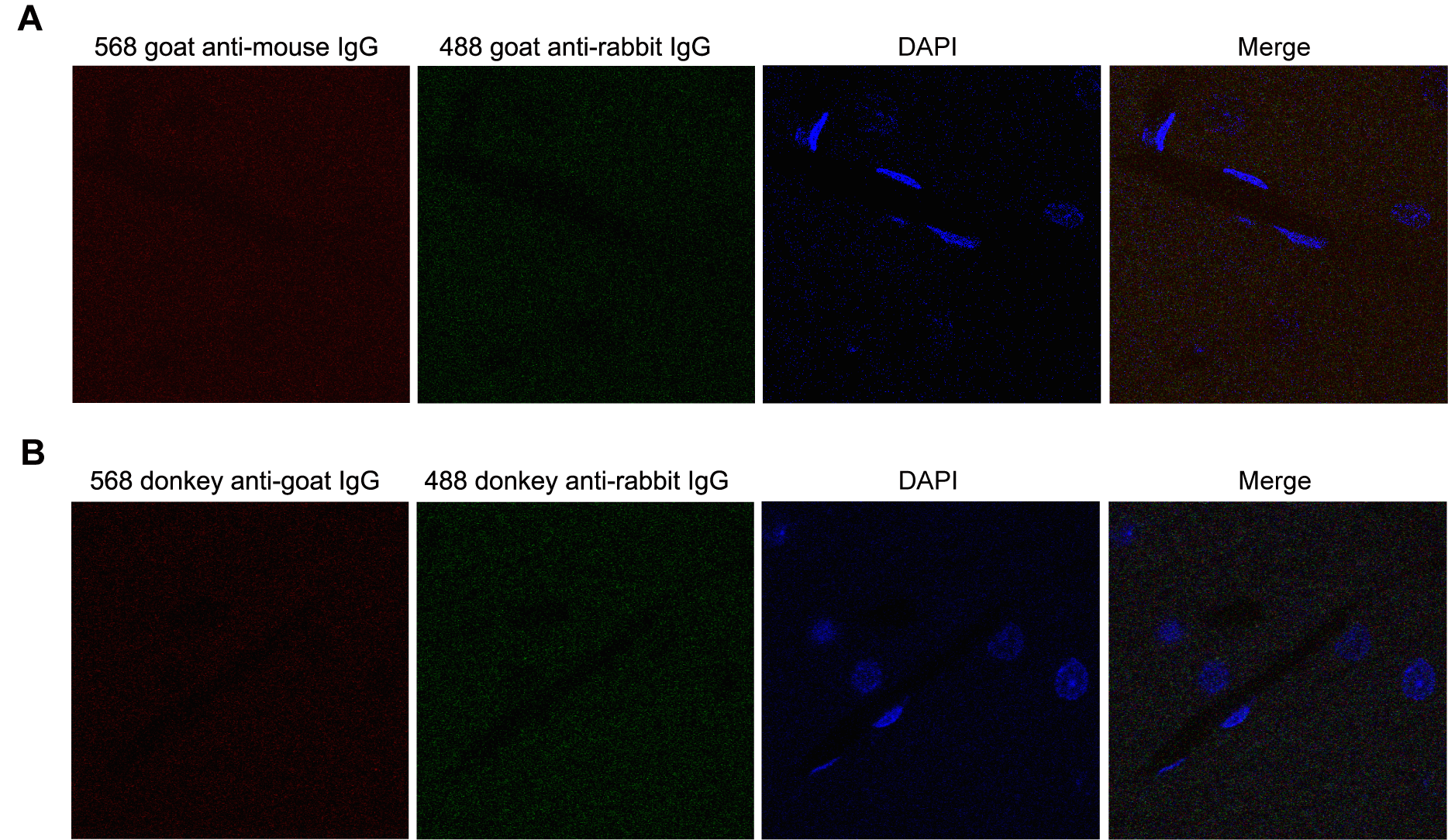

Supplement: Figure S1 — Negative controls for staining presented in Figure 1 . (A) Non-ischemic rat brain samples showing the absence of non-specific staining on using only goat secondary antibodies. From left to right: Alexa Fluor 568 goat anti-mouse IgG, Alexa Fluor 488 goat anti-rabbit IgG, 4′,6-diamidino-2-phenylindole (DAPI) stain, and a merged image. (B) Non-ischemic rat brain samples showing the absence of non-specific staining on using only donkey secondary antibodies. From left to right: Alexa Fluor 568 donkey anti-goat IgG, Alexa Fluor 488 donkey anti-rabbit IgG, DAPI stain, and a merged image. (TIF) [file pone.0098639.s001.tif]

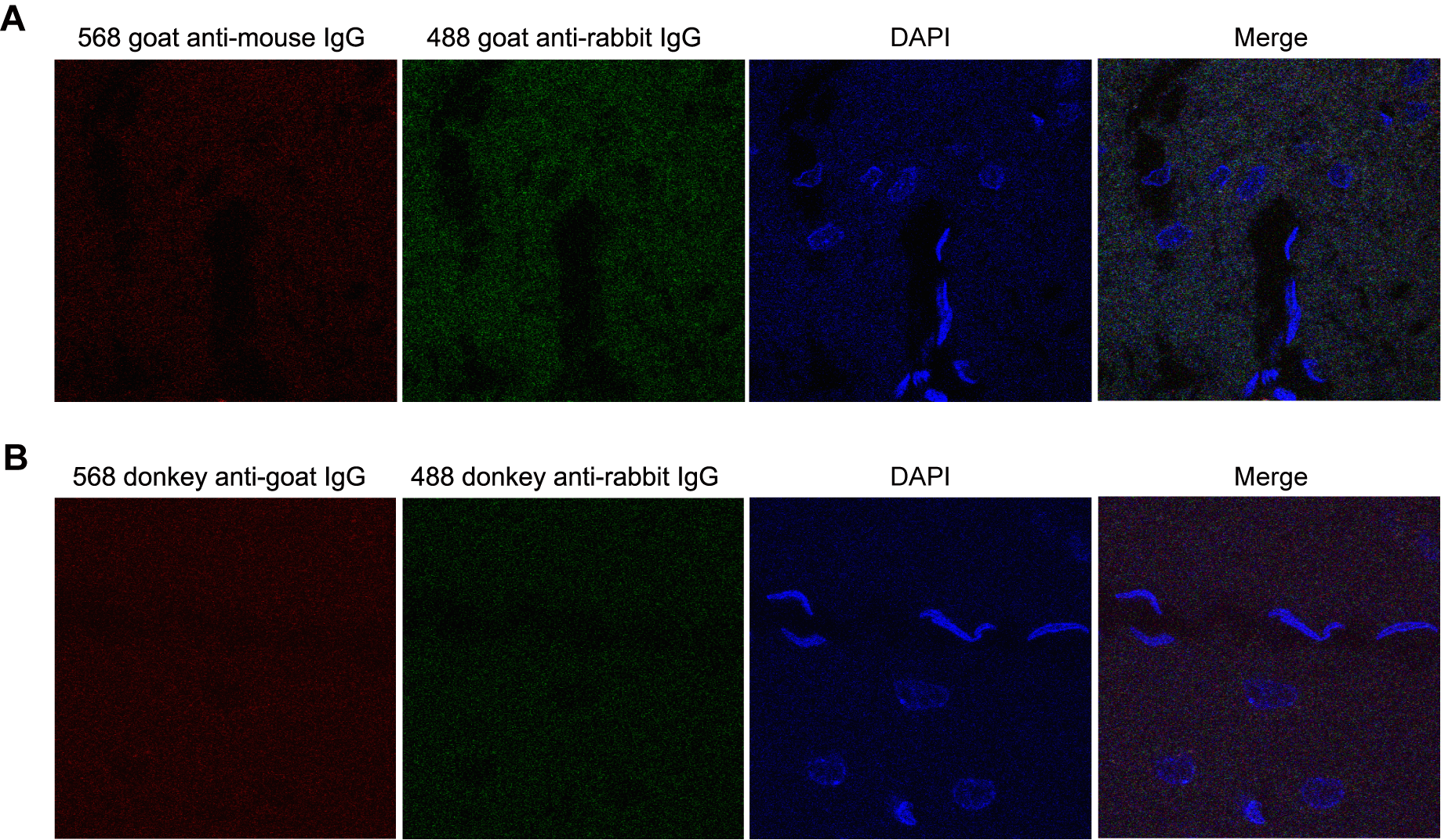

Supplement: Figure S2 — Negative controls for staining presented in Figure 4 . (A) Rat brain samples from cartilage oligomeric protein-angiopoietin-1 (COMP-Ang1) group showing the absence of non-specific staining using only goat secondary antibodies. From left to right: Alexa Fluor 568 goat anti-mouse IgG, Alexa Fluor 488 goat anti-rabbit IgG, 4′,6-diamidino-2-phenylindole (DAPI) stain, and a merged image. (B) Rat brain samples from COMP-Ang1 group showing the absence of non-specific staining using only donkey secondary antibodies. From left to right: Alexa Fluor 568 donkey anti-goat IgG, Alexa Fluor 488 donkey anti-rabbit IgG, DAPI stain, and a merged image. (TIF) [file pone.0098639.s002.tif]
